# Complement in the pathogenesis of Alzheimer’s disease

**DOI:** 10.1007/s00281-017-0662-9

**Published:** 2017-11-13

**Authors:** B. Paul Morgan

**Affiliations:** Systems Immunity Research Institute and Dementia Research Institute Cardiff, School of Medicine, Cardiff University, Cardiff, CF14 4XN UK

**Keywords:** Complement, Neurodegeneration, Alzheimer’s disease

## Abstract

The emergence of complement as an important player in normal brain development and pathological remodelling has come as a major surprise to most scientists working in neuroscience and almost all those working in complement. That a system, evolved to protect the host against infection, should have these unanticipated roles has forced a rethink about what complement might be doing in the brain in health and disease, where it is coming from, and whether we can, or indeed should, manipulate complement in the brain to improve function or restore homeostasis. Complement has been implicated in diverse neurological and neuropsychiatric diseases well reviewed elsewhere, from depression through epilepsy to demyelination and dementia, in most complement drives inflammation to exacerbate the disease. Here, I will focus on just one disease, the most common cause of dementia, Alzheimer’s disease. I will briefly review the current understanding of what complement does in the normal brain, noting, in particular, the many gaps in understanding, then describe how complement may influence the genesis and progression of pathology in Alzheimer’s disease. Finally, I will discuss the problems and pitfalls of therapeutic inhibition of complement in the Alzheimer brain.

## The brain as an island apart

The brain is without doubt a special organ, weight for weight the most energy-hungry, and isolated from peripheral insults by an intelligent barrier that dictates which substances enter and leave the brain parenchyma. This blood-brain barrier (BBB) comprises a collaboration between the specialised brain endothelium, vascular pericytes, perivascular glia and neurones, together forming a highly selective defensive wall to conserve brain homeostasis [1]. In the healthy brain, cell transit across this wall is extremely restricted; hence, peripheral immune cells are essentially excluded and transit of the majority of proteins from plasma, including complement proteins, limited such that levels in the brain parenchyma and cerebrospinal fluid (CSF) are typically between 1 and 0.1% those in plasma. A few proteins buck this trend either because they are actively transported across the BBB through receptor-mediated transcytosis (for example, transferrin) or are synthesised in the brain.

Important as the BBB is in maintaining homeostasis and protecting the brain from external and intrinsic insults, it is by no means a perfect barrier. Even in the healthy brain, there are regions where the BBB is compromised, particularly in the aged normal brain where evidence of barrier loss in and around the hippocampus has been described [2]. Whether these chinks in the brain’s armour are sufficient to allow complement protein access is untested. Almost any disease that affects the brain, and many systemic diseases, can trigger BBB leakage in various degrees. Numerous mediators have been implicated, including reactive oxygen species and activation of tissue metalloproteinases, but the dominant pathway to BBB breakdown is inflammation—central or systemic [3, 4]. Neurodegenerative and neuroinflammatory diseases are associated with disruption of the BBB, a consequence of endothelial leakage and inflammatory cell infiltration [5]. In multiple sclerosis (MS), a strongly inflammatory disease, individual areas of BBB breakdown are obvious and routinely imaged in gadolinium-enhanced MRI scans [6], but in slow burn, chronic inflammatory diseases like Alzheimer’s disease (AD), BBB impairment may be much more subtle, localised to areas of pathology and affecting specific transport processes—for example, transport of amyloid β [7]. Even in neuropsychiatric diseases, BBB impairment may occur and be a key driver of disease—for example, in schizophrenia, there is growing evidence that BBB leak contributes to perpetuation of the pathology [8]. Systemic inflammation, for example, as a result of infections or injuries, can independently cause disruption of the BBB and exacerbate barrier failure, perhaps explaining the well-documented impact of systemic illness on cognitive state in AD [4]. Indeed, in a mouse model of AD, the BBB was sensitised to disruption caused by low-dose lipopolysaccharide administration [9].

## Where does the healthy brain’s complement come from?

Complement provides an important innate immune defence against infection and essential contribution to effective garbage disposal in tissues; it is likely that complement plays these same homeostatic roles in the brain—but what is the source? The dominant source for most complement proteins in plasma (and tissues) is the liver; the exceptions to this rule are C1q, properdin and C7, synthesised predominantly in leukocytes, and factor D made in adipose tissue [10, 11]. With these exceptions, in most circumstances, hepatic-derived complement proteins secreted into plasma and leaching into tissues are the mediators of complement immune defence. However, the list of sites of extrahepatic synthesis of complement proteins has grown steadily and it is now clear that many organs and tissues can make most or all complement proteins locally. In most cases, this is at cottage-industry scale when compared to the liver mega-factory, routinely churning out grams per day of C3, and much more in response to acute phase triggers; however, in some circumstances, this local synthesis can be very significant. Indeed, studies in transplant recipients have suggested that around 10% of plasma complement proteins are derived from the various extra-hepatic sources [12]. The largest organ contributor to this extra-hepatic pool is likely the kidney; transplant studies have shown that a single-donor kidney can contribute ~ 5% of plasma C3 [13]. Locally produced complement may contribute to the circulating pool but, much more importantly, may provide local immune defence or drive pathology in that organ. In the kidney, this role for local complement has been abundantly demonstrated in experimental disease models by transplantation studies utilising complement-deficient organs and recipients.

In contrast to this clear evidence for renal complement synthesis, evidence for local synthesis of complement in the healthy brain is very limited—perhaps because brain transplantation remains unmastered! The ‘protected’ status of the brain, described above, implies that most plasma proteins are excluded from the healthy brain. The intact blood-brain barrier (BBB) will restrict or prevent access of complement proteins from the periphery; hence, local production may be particularly important for innate immune defence in the healthy brain. There have been numerous studies of complement protein expression in isolated brain cells and brain-derived cell lines. These data demonstrate that cell lines of microglial, astroglial and even neuronal origin can synthesise and secrete most or all complement proteins when appropriately stimulated in vitro [10, 11]. The relevance of these very artificial models to the situation in the normal healthy brain is tenuous at best. A handful of reports have described the identification of complement proteins or, critically in building the case for local production, message encoding complement proteins, in human brain tissue but mostly in pathological brain from inflammatory or degenerative cases [14–16]. One report compared expression of mRNAs for C1q, C3 and C4 in healthy and Alzheimer’s disease (AD) brain [17]. All three were expressed in healthy brain and levels of message were threefold higher in AD brain. Others described message encoding C1 subunits, C1 inhibitor (C1inh), C3 and C4 in healthy brain with expression increased 2–5-fold in Huntington’s disease (HD) brain [18]. Expression of complement regulators is low in the normal brain compared to other organs, with neurones particularly poorly endowed; in contrast, complement receptors, including the anaphylatoxin receptors, are expressed on glia and neurones, demonstrating a capacity to respond to local complement activation [19, 20].

Overall, a picture emerges of a low-complement environment in the healthy brain with little or no ingress from the circulation and minimal local biosynthesis; in this environment, brain cells are poorly protected from complement because they express relatively low levels of defence proteins, but, through expression of complement receptors, retain the capacity to respond to any complement activation that does occur locally. In the inflamed or injured brain, the situation is likely very different.

## What is complement doing in the normal brain? Keeping house and tending connections

**Fig. 1:**
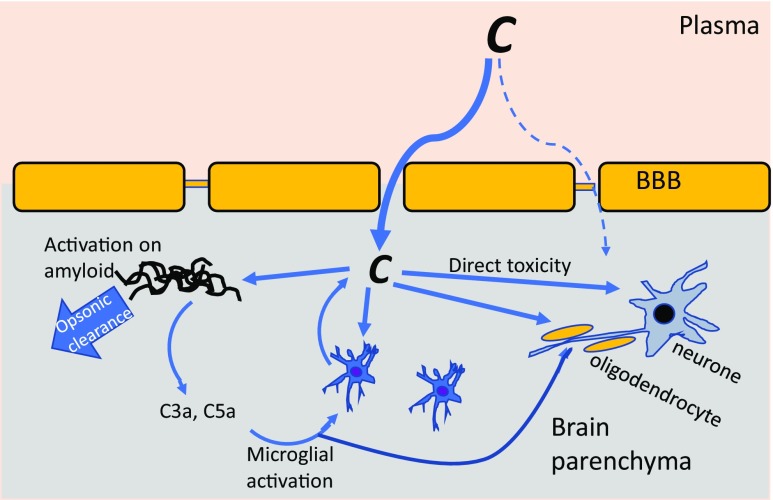
Complement dysregulation in the AD brain. Complement (C) may access the brain parenchyma from plasma through a disrupted BBB or may be made locally by glia and/or neurones. Complement is activated (via the CP) on amyloid plaques; this may facilitate opsonic clearance but also drives inflammatory activation of glia via C5a (and C3a). Activated microglia synthesise inflammatory cytokines and more complement proteins, stokeing the flames. Complement activation on neurones and oligodendrocytes leads directly to cell damage and death. Injured and dead cells activate more complement, leading to dysregulation, further inflammation and tissue damage

In the periphery, complement functions to protect against infections and dispose of garbage (Fig. 1a). In the brain, complement likely performs these same roles but also contributes to maintenance and homeostasis in other ways. Clues to these other roles have emerged from detailed analyses of complement-deficient mice. In C3-deficient mice, removal of synapses from damaged neurones is impaired, synapse number is increased and cognitive performance is enhanced [21, 22]. Ageing-associated loss of synapses in the hippocampus was also reduced in C3-deficient mice and associated with improved learning and memory, suggesting that complement is bad for synapse health in ageing [23]. However, too many synapses are not entirely a good thing: C1q-deficient mice showed defective synaptic pruning, resulting in an over-connected brain and a remarkable susceptibility to epileptic events [24–26]. Together, the data demonstrate an essential homeostatic role of complement activation in the normal brain to tag damaged or effete synapses and facilitate the continuing re-wiring that occurs in development and throughout life as part of brain plasticity. Several neurodevelopmental and neurodegenerative diseases, including schizophrenia and AD, have been associated with abnormal synaptic pruning; the relevance of complement dysregulation in these diseases is the subject of current research and thoroughly reviewed elsewhere [27].

## What is so special about the brain? Demolishing the blood-brain barrier

Although the BBB is not absolute, and some macromolecules bypass the barriers [28], complement proteins without exception cannot penetrate to reach the brain parenchyma unless BBB integrity is disrupted. Almost any disturbance of brain homeostasis—infection, injury, infarct or inflammation—can bring down to the barrier to some degree. As noted above, systemic factors, including infections and inflammation, can also compromise the BBB [3]. Whatever the trigger, barrier disruption allows ingress of normally excluded proteins, including complement proteins, into the parenchyma surrounding the initiating damage. Injured, ischaemic, apoptotic or foreign surfaces will trigger activation of complement, causing further damage, particularly on cells poorly protected by regulators, and driving a vicious cycle of inflammation, further BBB disruption and influx of more complement proteins to feed the flames (Fig. 1). This scenario is most clearly enacted in the case of ischaemic stroke where reperfusion of the ischaemic tissue causes local inflammation and loss of BBB integrity; influx of complement proteins and activation around the infarct causes lesion expansion. This critical role of complement is clearly demonstrated in stroke models where complement deficiency or anti-complement drugs markedly reduce lesion size [29, 30].

**Fig. 2:**
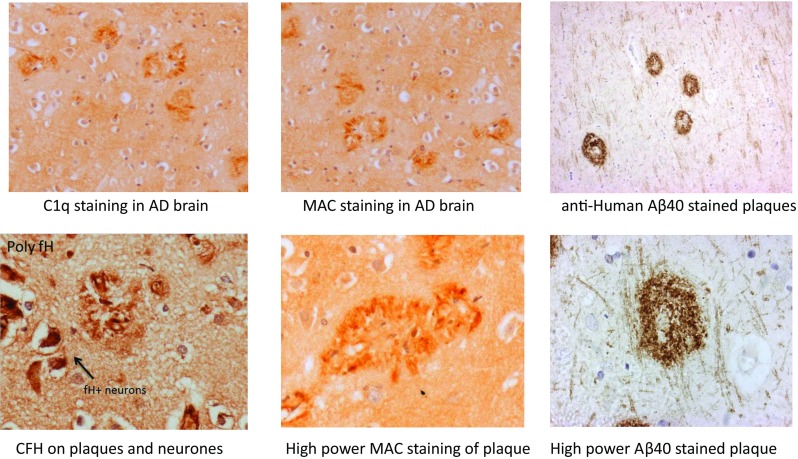
Complement components and activation products in the AD brain. Examples of AD brain sections stained with different complement antibodies: plaques stain strongly for C1q, MAC and CFH. Neurones are also strongly positive for CFH. Aβ40 staining of plaques is shown as a control

In many other brain pathologies, evidence that complement is activated at some stage in the disease process is clear. In MS, complement activation products are abundant in and around lesions, likely reflecting BBB breakdown and influx, although increased local biosynthesis may also contribute [31, 32]. In AD, complement activation products richly decorate plaques and tangles [33] (Fig. 2). Of course, association does not imply causation; the presence of complement activation products in areas of pathology in advanced disease could represent a secondary phenomenon unrelated to the disease process. However, taken together with other lines of evidence, a strong case can be developed for a causal role of complement in many brain diseases. The case for AD is expanded below.

## Complement in neurodegeneration

Neurodegenerative diseases are defined by the progressive loss of neurons and so have been considered incurable and untreatable. Mitochondrial dysfunction, protein missfolding and other forms of systems failure underpin the pathology in most neurodegenerative diseases. Global neurodegeneration causes progressive dementia, as seen in AD. In Parkinson’s disease, the earliest pathology affects the substantia nigra leading to the typical movement disorder, but global disease and dementia follows. In amyotrophic lateral sclerosis (ALS) and other motor neurone diseases, motor neurones are lost selectively leading to increasing disability but with retention of cognitive function (as Stephen Hawking amply demonstrates).

AD is the commonest cause of dementia with over 850,000 sufferers in the UK alone, over 50 million globally and an economic cost expected to hit $1 trillion in 2018 (statistics from Alzheimer’s Research UK; http://www.alzheimersresearchuk.org/). Incidence is increasing as the population ages, signalling a global epidemic of unparalleled magnitude. From its first description in 1906 until very recent times, AD was considered a purely degenerative disease, characterised by progressive loss of neurones and brain atrophy [34]. The first suggestions that AD might have an inflammatory component emerged in the 1980s; acute phase reactants were shown to be increased in plasma from AD patients compared with controls, glial cells in AD brain showed changes associated with inflammation, and prior use of non-steroidal anti-inflammatory drugs (NSAIDs) for unrelated conditions protected against AD, findings replicated in animal models [35]. A review published in 1994 concluded that the evidence studies implicating inflammation in AD was strong and made the case for formal trials of anti-inflammatory therapies [36]; surprisingly, more than 20 years later, there have still been no large trials of the impact of NSAIDs or other anti-inflammatory agents administered early in the disease process on the course of AD. Importantly, inflammatory activation of microglia and astrocytes is now recognised as a key marker of the AD brain [37, 38].

As evidence accumulates that AD is an inflammatory disease associated with some degree of BBB breakdown, it becomes an inevitability that complement will play a role in the pathology. Complement is a strongly pro-inflammatory system with the capacity to cause damage to self both indirectly through recruitment and activation of immune cells and directly through its cytotoxic effector, the membrane attack complex (MAC). Complement causes pathology when regulation fails; this leads to excessive and widespread activation, driving inflammation and tissue damage. Acute dysregulation can cause overwhelming injury, as seen in sepsis; in most diseases, chronic, low-grade dysregulation occurs that leads to the accumulation of damage over time.

The nervous system is particularly susceptible to damage caused by complement dysregulation because its resident cells, neurones and glia are poorly protected from the products of complement activation [19, 20, 39]. Degrees of dysregulation that might be tolerated in other tissues thus cause damage in the brain and other parts of the nervous system. Potential triggers for complement dysregulation in neurodegeneration are legion. Autoantibodies can initiate the classical pathway; protein aggregates, damaged cells, exposed myelin antigens or pathogens can trigger multiple activation pathways; trauma or anoxia activates complement locally or globally (Fig. 1). This perfect storm can drive a vicious cycle of complement activation and inflammation; restoring complement homeostasis may break this cycle and ameliorate disease.

## Immunohistochemistry implicates complement in AD

A role for complement as a driver of inflammation in the AD brain first emerged in the 1980s, based upon guilt by association; immunohistochemistry in late-stage AD brain demonstrated that the prototypical lesions, amyloid plaques, were richly decorated with complement proteins [40–43]. Early components of the classical pathway (C1q, C4, C1inh) were particularly abundant, but detection of terminal pathway proteins was inconsistent [44, 45]. Importantly, staining with specific antibodies demonstrated the presence of complement activation products including C3b/iC3b and the terminal complement complex (TCC), confirming that the complement system was activated in and around plaques in AD (Fig. 2). Of particular note, dystrophic neurites were TCC/MAC-positive, suggesting that MAC may cause direct damage, contributing to neuritic dystrophy and neuronal loss in AD brain [46]. Of course, immunohistochemistry in post-mortem brain only provides information on end-stage disease and gives no clues as to whether complement activation occurs early in the disease process. In unpublished studies, we examined complement protein deposition in post-mortem brain at different stages of AD, from the earliest (Braak stage I) to late (Braak stage V), and showed that even in early disease, complement was present and activated in areas of pathology. C1q appears to be an important marker of neurodegeneration in both rodents and man; however, C1q staining is also associated with brain ageing in the absence of dementia [47]. C1q staining also strongly correlated with pathology in MS brain, with predominant staining for both C1q and the regulator C1inh on neurones [48]. Taken together, the evidence suggests that complement is activated in areas of pathology at all stages of neurodegeneration. As noted above, the source of complement within the brain in health and disease is debated, but there is considerable evidence supporting local synthesis of complement proteins in the brain in neurodegeneration. In MS, glia and neurones generate C1q, C3 and other key components [48], and it has been reported that cerebral vascular endothelial cells can make all complement proteins [49]. These latter authors showed that endothelial complement biosynthesis was increased by exposure ex vivo to aggregated Aβ and suggested a role for endothelial complement in forming the characteristic amyloid deposits in vessel walls in AD brain, cerebral amyloid angiopathy.

## Evidence from animal models implicates complement in AD

Animal models have been both a blessing and a curse for AD research. For rare, inherited dementias, including several of the early-onset forms of AD, single genes have been implicated and here, animal models replicating the genetic change and (to varying degrees) the pathological course can be generated and inform understanding [50]. For the much more common late-onset AD, animal models are less informative and often misleading. Late-onset AD is genetically complex and represents a spectrum of disease, not a single, homogeneous condition. There are multiple pathological hallmarks, including but not restricted to the classical amyloid plaques and tau tangles, and large inter-individual variability in pathological changes and disease course. Despite these many issues, a huge amount of work has been performed to generate and characterise rodent models of AD that replicate some of the pathological and clinical aspects of the disease [51, 52]. Evidence from rodent models has in many instances informed understanding of human AD, but has also occasionally misled.

Much of the immuohistochemical evidence for complement dysregulation noted above in humans has been recapitulated in various animal models; for example, in a PS1/APP mouse model, C1q was co-localised with amyloid plaques [53]. Roles of complement in mouse AD models have been tested either using complement-deficient mice or administration of anti-complement drugs. The resulting data has been confusing and inconsistent, reflecting the variability in models noted above and reviewed elsewhere [54]. In two related models, Tg2576 and APPPS1 (Tg2576 x mutant PS1), both characterised by increased Aβ plaques, activated microglia and astrocytes and dystrophic neurites, C1q deficiency was strongly protective, supporting a role for classical pathway activation [55]. In sharp contrast, in a related APP-transgenic (hAPP) model, C3 deficiency was associated with increased plaque burden and neuronal loss in aged mice [56]. In support of this latter finding, inhibition of complement activation by expression of the C3 convertase regulator soluble Crry in the brain exacerbated amyloid plaque formation and neuronal degeneration in the hAPP model [57]. These authors first showed that overproduction of TGF-β1 in the model was associated with increased microglial activation, reduced plaque load and elevated C3 levels in brain. Together, these studies were interpreted as demonstrating a role for C3 in the clearance of plaques and maintenance of neuronal viability. A recent study testing effects of C3 deficiency in the APPPS1 model emphasised the dual-edged nature of complement; C3-deficient mice showed an increase in amyloid plaque load, in agreement with other studies, but were nevertheless protected against cognitive decline [58]. There were fewer plaque-associated microglia and astrocytes, and inflammatory cytokine levels were lower in brain, suggesting that the microglial phenotype was markedly different in the absence of C3.

A recent report linked the ‘housekeeping’ synaptic processing roles of complement described above with complement roles in AD. In the hAPP model, C1q labelling of synapses was seen early and in much higher amounts than in normal mice; inhibition of C1q using a blocking antibody or deficiency of either C1q or C3 reduced synaptic elimination and improved hippocampal function [59]. Notably, blockade of microglial CR3, the phagocytic receptor for the complement opsonin iC3b, also protected synapses in the model, demonstrating that synaptic removal in this context involved a collaboration between complement and microglia.

## Genetics implicates complement in AD

The most convincing evidence that complement is *causatively* involved in AD comes from genetics. The most significant genetic risk factor for late-onset AD is the e4 allele of the ApoE lipoprotein; this is associated with increased brain amyloid burden through mechanisms that remain unclear. Of the handful of other genes linked to AD in recent genome-wide association studies (GWAS), several are complement-related—the genes encoding clusterin and complement receptor 1 (CR1) were linked in first studies [60, 61], and more complement genes emerged from pathway studies, notably the genes encoding C1s and C9 [62–64]. Precisely how these complement genes and pathways link to AD pathogenesis is the subject of intense research and debate; an emerging consensus suggests roles in waste disposal and inflammation are keys.

Clusterin, also known as ApoJ, is a multifunctional molecular chaperone that, among its many activities, is a fluid phase regulator of the complement terminal pathway. The clusterin (*CLU*) gene is the third most associated risk gene for late-onset AD. In GWAS, three single-nucleotide polymorphisms (SNPs) in the *Clu* gene, rs11136000 (intronic), rs2279590 (intronic) and rs9331888 (non-coding) were significantly associated with AD in a predominantly Caucasian cohort [60, 61]. All three SNPs are non-coding/intronic and little is known regarding how these variants impact clusterin protein or the development of AD pathology. Numerous studies have addressed roles of the *Clu* SNP in Aβ deposition and plaque assembly, neuronal health and metabolism, lipid handling and effects on brain imaging or biomarkers. All three SNPs impacted the amount of Aβ deposition, while the rs9331888 SNP increased rate of Aβ deposition, and rs9331888 was associated with hippocampus volume, all assessed by imaging [65]. The rs11136000 SNP associated with CSF Tau levels in AD patients [66]. These authors also described an intracellular form of clusterin in AD model mice and humans, showed its association with the AD risk *Clu* SNP and that intracellular clusterin interacted with another GWAS hit, BIN1, to drive Tau pathology in AD, thereby identifying a pathway that linked two genetic associations.

**Fig. 3:**
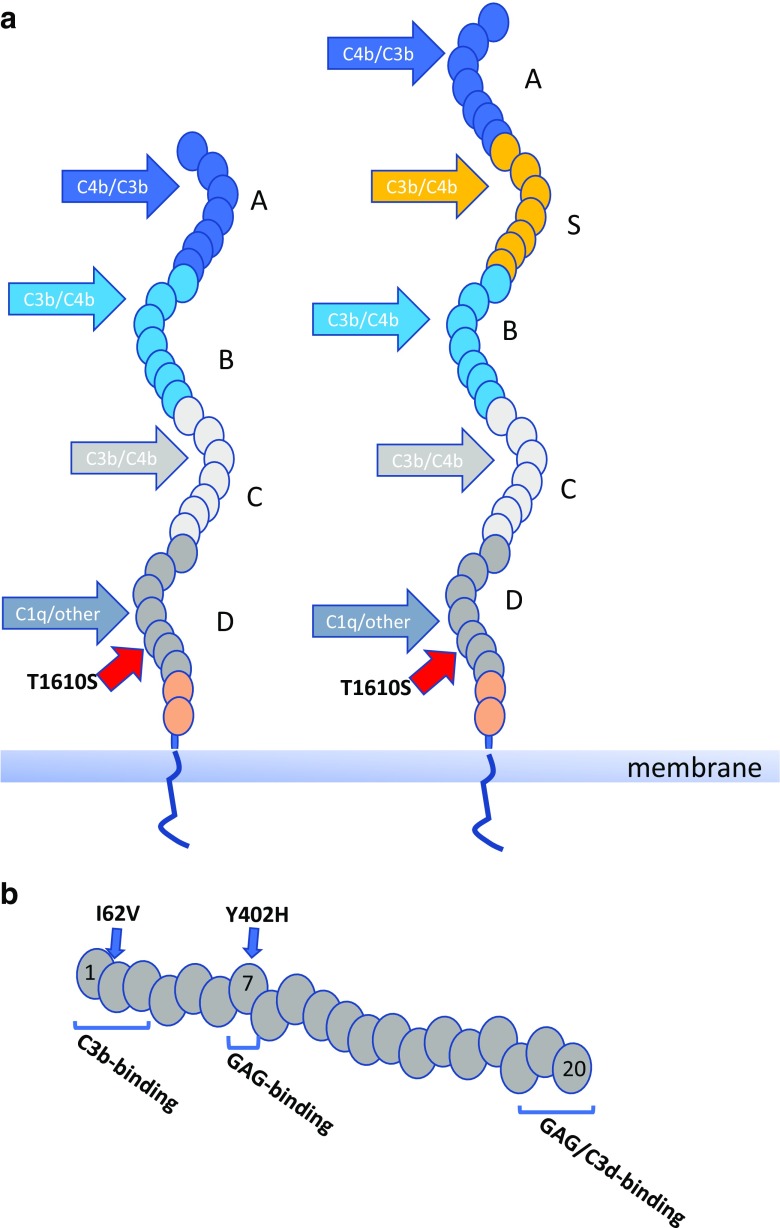
Association of SNPs in *CR1* and *CFH* with AD. **a** The short (CR1*1) and long (CR1*2) variants of CR1 are illustrated, differing by the acquisition of an extra LHR (LHR-S) in the long forms; CR1*2 is strongly associated with the risk allele at the rs6656401 SNP. Individual SCRs are represented by ovals. Binding sites for C3b, C4b and CR1 are indicated. The T1610S (rs4844609) coding SNP in the 26th SCR is adjacent the C1q binding site in LHR-D. **b** CFH comprises a string of 20 SCRs. Binding sites for C3b, C3d and glycosaminoglycans (GAGs) are indicated. The two coding SNPs associated with AD in some ethnic groups respectively cause single amino acid changes I62V (at the interface of SCRs 1 and 2 and in the C3b binding site) and Y402H (in SCR 7 and part of a GAG binding site)

CR1 is the cell surface receptor for the C3b fragment; CR1 on erythrocytes plays important roles in immune complex transport and phagocytic cell expression supports phagocytosis of complement opsonised particles [67]. GWAS identified two SNPs associated with AD; rs4844609, a coding SNP that causes a single amino acid change (T1610S) in the 26th short consensus repeat (SCR) of CR1, a region reported to be a C1q binding site, and an intronic SNP, rs6656401, that is very strongly associated with the CR1 length polymorphism [61] (Fig. 3). Increased binding affinity for C1q has been reported for the risk allele of rs4844609 [68]. The long form of CR1 (CR1*2) that is associated with the risk allele at the rs6656401 SNP differs from the more common shorter CR1*1 in that it has acquired an additional long homologous repeat (LHR); each LHR comprises seven SCRs, each homologous to its equivalent in the other LHRs. CR1*1 extracellular domain comprises 4 LHRs while CR1*2 comprises 5. The additional LHR adds C3 fragment binding sites so can be considered a gain-of-function; however, CR1*2 is associated with a lower copy number of CR1 on erythrocytes sites, and perhaps other cells [69]. Indeed, it has been suggested that reduced CR1 expression on erythrocytes leading to impaired amyloid clearance is the mechanism by which the rs6656401 SNP impacts AD pathology [70].

Screening for AD-associated genes in a large Chinese cohort demonstrated rather weak association with the most significant hits in the Caucasian analyses and identified strong associations with SNPs in the gene encoding the complement regulator factor H (CFH); the two strongest associating SNPs, rs1061170 and rs800292, are both coding variants causing respectively a H402Y and a I62V change in the protein [71] (Fig. 3). In both cases, the risk allele was associated with higher atrophy rate and more severe cognitive decline. Of note, these two CFH SNPs have previously been reported as strong risk factors for age-related macular degeneration in diverse cohorts and shown to affect the complement regulatory activity of CFH [72–74].

## Complement biomarkers and AD

There is a lack of informative biomarkers to aid diagnosis, stratification or prediction of outcome in AD or that predict progression from mild cognitive impairment (MCI) to AD. Biomarkers could be measured in CSF or plasma; however, sampling CSF requires lumbar puncture—invasive and potentially dangerous—and certainly not a viable option for a screening test in the healthy elderly. A few plasma markers have been described but are untested in preclinical disease and likely unsuitable for early diagnosis [75, 76]. The goal for current studies is to deliver a highly informative plasma biomarker or set of markers that enable early diagnosis and predict disease course. If complement dysregulation is a feature of AD, then measurement of complement biomarkers might help diagnose, predict or stratify the disease. Among the complement proteins reported to be associated with AD, CFH and clusterin, both implicated from genetics, emerge from multiple studies, although there are conflicting reports that do not support these associations. Plasma clusterin levels were associated with disease, disease subtype and rate of progression [77–79], and plasma factor I (CFI) levels were predictive of brain atrophy [80].

**Fig. 4:**
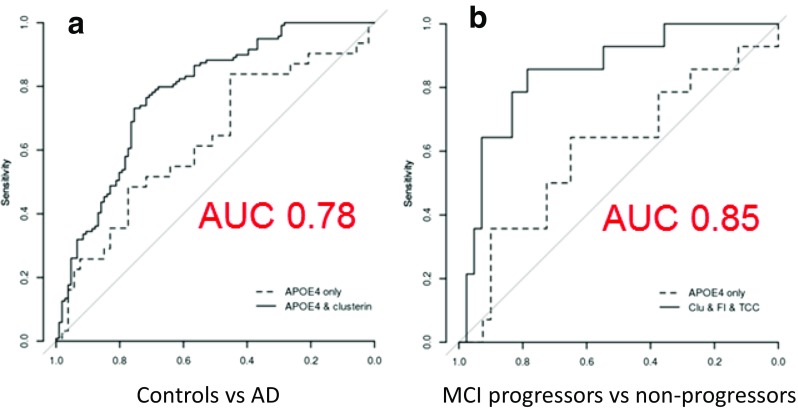
Plasma complement biomarkers predict progression in mild cognitive impairment (MCI). Results of a multivariate analysis to identify the best plasma complement biomarkers to differentiate AD from controls (**a**) and to predict progression in individuals with MCI (**b**). Only clusterin was significant in the first analysis; with ApoE status, it gave a predictive power (AUC) of 0.78. Clusterin, CFI and TCC were together highly predictive of progression in MCI (AUC of 0.85). Modified from Reference [81]

We measured a complement marker set comprising five complement proteins and four activation products in plasma from MCI, AD and controls [81]. Assessed as single analytes, only clusterin differed significantly between controls and AD and when combined with relevant co-variables was highly predictive of disease. When complement analytes were measured in MCI, 3 (clusterin, CFI, TCC) were different between those who a year later had converted to AD and those who did not convert; a multivariate model based on these analytes was highly predictive of risk of progression in MCI individuals who had converted to dementia 1 year later compared to non-converters; a model combining these three analytes was highly predictive of conversion with a predictive power of 85% (Fig. 4). We also correlated plasma biomarkers with genetic risk of AD measured using a polygenic score that took account of all known genetic risk factors [82]. The strongest association was again with clusterin, higher levels in plasma correlating strongly with polygenic score. Our current aim is to develop a ‘best set’ of complement and other inflammatory markers in plasma that can be used to build multi-parameter models for disease prediction and stratification for therapy in MCI and AD.

## Targeting complement in AD

The text above makes the case that complement is an important contributing factor to inflammation and neurodegeneration in AD. Although complement may not be the primary trigger for the pathology, once activated, it is a powerful driver of the disease, a situation exacerbated by the sensitivity of brain cells to complement-mediated damage. However, the evidence suggests that the situation in AD is more complex with complement also having beneficial effects by limiting the accumulation of debris, an activity that may be the predominant contribution early in the disease process. Any attempt to target complement activation in AD must take account of this complexity and requires signposts from biomarkers or imaging to identify whether, when and how complement should be targeted. The first two of these considerations await clarification from biomarker and imaging studies; I will here focus on the last—how to target complement dysregulation in the AD brain.

As comprehensively detailed elsewhere in this volume (Harris CL, Expanding horizons in complement drug discovery: challenges and emerging strategies), complement therapeutics is undergoing a sea change with numerous new drugs appearing targeting different stages and effectors. No one anti-complement agent will fit all disease requirements and selection of an appropriate agent requires an understanding of the nature of the dysregulation—which pathways are activated, which of the activation products are causing damage and which are irrelevant or even protective. In AD, choice of therapy will also be dictated by factors such as ease of administration, suitability for long-term use, safety and, of course, cost. This last factor is of particular relevance for anti-complement therapies since currently available drugs are eye-wateringly expensive and their use in a common, chronic disease like AD would be unaffordable in most health systems [83]. Only two anti-complement agents are currently licenced for use, plasma-derived C1 esterase inhibitor (C1INH; Cinryze™; Berinert™) and the C5-blocking monoclonal antibody Eculizumab™. C1INH is a large (~ 70 kDa) serine protease inhibitor, developed for treatment of hereditary angioedema and untested in neurological diseases; it inhibits the complement classical pathway by dissociating the C1 complex. Eculizumab, licenced for use in two rare non-CNS conditions, has been tested in a small trial in the rare demyelinating disease neuromyelitis optica with good effect [84]. Eculizumab prevents cleavage of C5, thus stopping generation of the effectors C5a and MAC. Neither of these agents is a likely candidate for testing in AD, primarily because they will not cross the BBB.

**Fig. 5:**
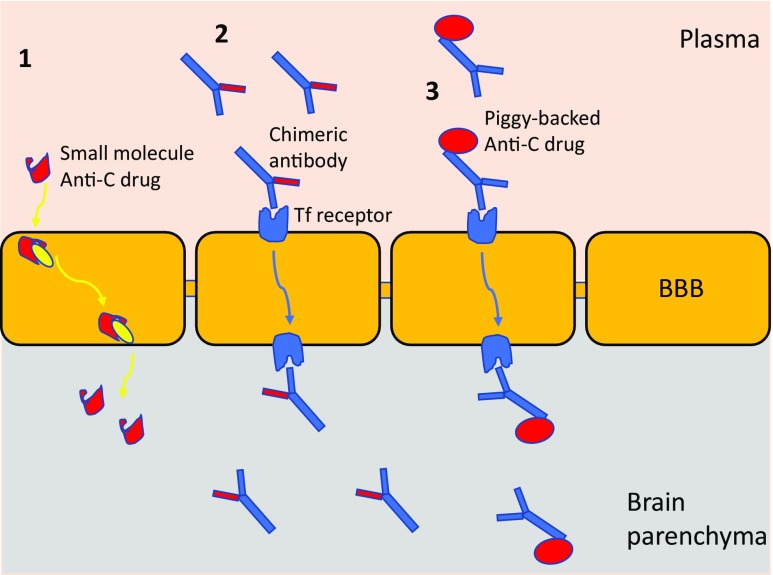
Getting anti-complement drugs into the AD brain. Carrier-mediated transport (CMT) actively delivers some small-molecule drugs into the brain (1), utilising one of a large number of intracellular transporters. A small-molecule anti-complement drug (illustrated in red) could be designed to engage CMT for delivery across the BBB. For larger molecules, ‘Trojan Horse’ methods can be utilised to deliver, hijacking receptor-mediated transport (RMT) systems. Chimeric antibodies capable of binding a relevant receptor (usually either the transferrin receptor or the insulin receptor) through one arm and carrying an anti-complement site on the other (in red; anti-C1q; anti-C5 etc) will be transported across the BBB and released into the brain parenchyma to inhibit complement. Alternatively, a complement regulator can be coupled recombinantly or chemically to the anti-receptor antibody (3), allowing it to piggy-back across the BBB

For a typical monoclonal antibody or other large protein drug administered systemically, brain levels are around 0.1% of those in the blood, making it essentially impossible to get enough into the brain to inhibit a relatively abundant complement target. Drug delivery then becomes the biggest barrier to treating AD. To circumvent this issue, either drugs must be designed that are BBB permeant (generally small, lipophilic entities) or ‘Trojan horse’ carrier methods must be used [85] (Fig. 5). The molecular weight threshold for effective drug delivery across the BBB is ~ 400 Da [86]; hence, most drugs labelled as ‘small molecules’ will not readily access the brain. Lipid solubility inversely correlates with hydrogen binding capacity because more hydrogen bonds translates to more water-binding, effectively increasing the molecular weight of the drug [87]. Medicinal chemistry approaches to create BBB-penetrant drugs by increasing lipophilicity have met with mixed success. Some small-molecule drugs are actively transported into the brain, a process termed carrier-mediated transport (CMT); for example, DOPA used in Parkinson’s disease. There are numerous CMT pathways across the BBB, offering considerable potential for drug delivery. For larger drugs such as antibodies, receptor-mediated transport (RMT) systems can be exploited; hijacking brain endothelial cell transporter receptors for insulin or transferrin (Tf). Much effort has been expended to modify anti-amyloid antibodies to bind these receptors, for example, by piggybacking onto anti-receptor antibodies [88]. Similar approaches have been used to deliver TNF inhibitory proteins for therapy of AD [89] (Fig. 5).

The design of anti-complement agents for efficient delivery to the brain has yet to begin. Chimeric versions of Eculizumab or other anti-complement antibody therapeutics that can engage RMT pathways across the BBB should be an early priority. Annexon Biosciences (http://www.annexonbio.com/science/) have developed C1q-blocking monoclonal antibodies (ANX005/007) for therapy of AD but have not yet addressed the delivery problem. Another anti-C1q antibody was neuroprotective in a mouse peripheral neuropathy model [90]. Numerous small-molecule anti-complement drugs targeting complement receptors (for example, C5a receptor antagonist peptides; [91]) or complement enzymes (for example, factor D blockers; [92]) are in development and approaching the clinic; however, to date, none have been designed with BBB penetrance in mind. This is a crucial gap that needs to be closed to enable targeting of complement dysregulation in the brain in AD.

## Concluding remarks

Over the last decade, there has been an explosion of understanding regarding the relevance of inflammation in AD and other neurodegenerative diseases. Alongside this, the roles played by the potent pro-inflammatory and cytotoxic system, complement, have been recognised. Complement plays complex roles in brain homeostasis and likely has both protective and exacerbating effects on disease. Evidence suggests that complement restricts amyloid plaque formation and aids clearance of plaque components, but also contributes to the switch of microglia and astrocytes into activated neurotoxic cells that drive the pathology [93]. Given this complexity, anti-complement therapies need to be given to the right patients, at the right time, target the right pathway and get to the right place, a host of challenges that have yet to be addressed. Biomarkers and imaging to stratify and select will be key to the success of any therapeutic intervention, though perhaps the biggest challenge is to create anti-complement drugs that are brain-permeant and can get to the sites of disease even in the early stages. Properly constituted clinical trials of appropriate anti-complement therapies are now urgently needed. Evidence to date suggests that agents specifically inhibiting classical pathway activation might be most appropriate, although C5a antagonists or inhibitors of AP activation might also be useful (and perhaps less risky). Protocols to monitor systemic and central complement inhibition and minimise risk of infection or other iatrogenic effects are needed to support future trials. Despite these many issues, complement represents a tractable target in a currently untreatable disease.
