# Correction to: Complement in the pathogenesis of Alzheimer’s disease

**DOI:** 10.1007/s00281-018-0709-6

**Published:** 2018-09-21

**Authors:** B. Paul Morgan

**Affiliations:** Systems Immunity Research Institute and Dementia Research Institute Cardiff, School of Medicine, Cardiff University, Cardiff, CF14 4XN UK

**Correction to: Semin Immunopathol (2018) 40:113–124**

10.1007/s00281-017-0662-9

The presentation of Fig. 2 was incorrect. The correct version of Fig. 2 is given below.

**Fig. 2:**
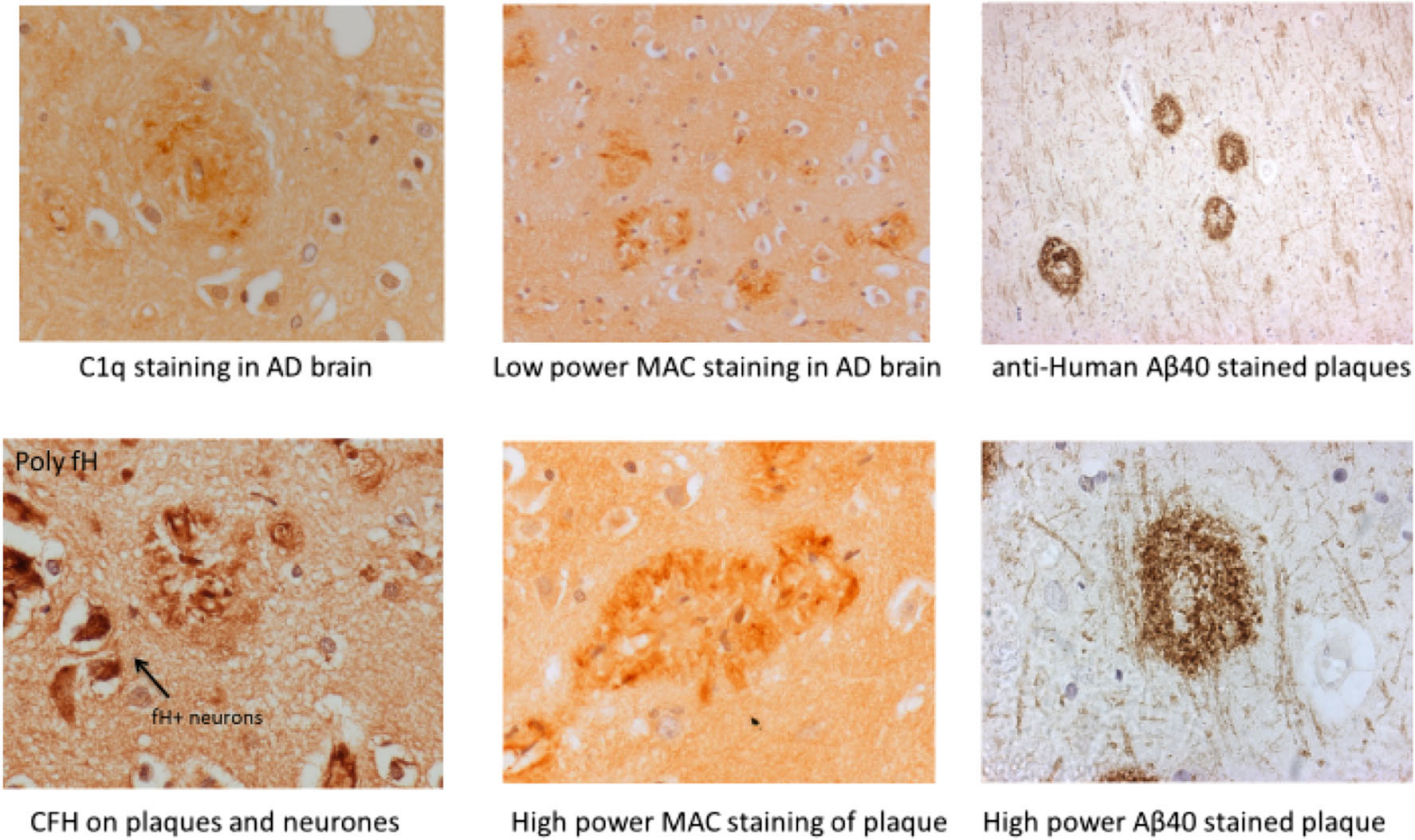
Complement components and activation products in the AD brain. Examples of AD brain sections stained with different complement antibodies: plaques stain strongly for C1q, MAC and CFH. Neurons are also strongly positive for CFH. Aβ40 staining of plaques is shown as a control

**Open Access** This article is distributed under the terms of the Creative Commons Attribution 4.0 International License (http://creativecommons.org/licenses/by/4.0/), which permits unrestricted use, distribution, and reproduction in any medium, provided you give appropriate credit to the original author(s) and the source, provide a link to the Creative Commons license, and indicate if changes were made.

